# Uptake of breast cancer preventive therapy in the UK: results from a multicentre prospective survey and qualitative interviews

**DOI:** 10.1007/s10549-018-4775-1

**Published:** 2018-04-24

**Authors:** Julia Hackett, Rachael Thorneloe, Lucy Side, Michael Wolf, Rob Horne, Jack Cuzick, Samuel G. Smith

**Affiliations:** 1 Leeds Institute of Health Sciences, University of Leeds, Worsley Building, Clarendon Way, Leeds, LS2 9NL UK; 2 Wessex Clinical Genetics Service, University Hospitals Southampton, Southampton, UK; 3 UCL Institute for Women’s Health, London, UK; 4 Division of General Internal Medicine and Geriatrics, Northwestern University, Chicago, USA; 5 Centre for Behavioural Medicine, School of Pharmacy, University College London, London, UK; 6 Wolfson Institute of Preventive Medicine, Queen Mary University of London, London, UK

**Keywords:** Preventive therapy, Chemoprevention, Breast cancer, Decision-making, Medication

## Abstract

**Purpose:**

Uptake of preventive therapy for women at increased breast cancer risk in England is unknown following the introduction of UK clinical guidelines in 2013. Preventive therapy could create socioeconomic inequalities in cancer incidence if it is more readily accepted by particular socio-demographic groups. In this multicentre study, we investigated uptake of tamoxifen and evaluated socio-demographic and clinical factors associated with initiation. We explored women’s experiences of treatment decision-making using qualitative interview data.

**Methods:**

Between September 2015 and December 2016, women (*n* = 732) attending an appointment at one of 20 centres in England to discuss breast cancer risk were approached to complete a survey containing socio-demographic details and nulliparity. Of the baseline survey respondents (*n* = 408/732, 55.7% response rate), self-reported uptake of tamoxifen at 3-month follow-up was reported in 258 (63.2%). Sixteen women participated in semi-structured interviews.

**Results:**

One in seven (38/258 = 14.7%) women initiated tamoxifen. Women who had children were more likely to report use of tamoxifen than those without children (OR = 5.26; 95%CI: 1.13–24.49, *p* = 0.035). Interview data suggested that women weigh up risks and benefits of tamoxifen within the context of familial commitments, with exposure to significant other’s beliefs and experiences of cancer and medication a basis for their decision.

**Conclusions:**

Uptake of tamoxifen is low in clinical practice. There were no socio-demographic differences in uptake, suggesting that the introduction of breast cancer preventive therapy is unlikely to create socioeconomic inequalities in cancer incidence. Women’s decision-making was influenced by familial priorities, particularly having children.

**Electronic supplementary material:**

The online version of this article (10.1007/s10549-018-4775-1) contains supplementary material, which is available to authorized users.

## Introduction

Breast cancer is the most common female cancer in the UK [1], and incidence is projected to rise over the next 20 years [2]. A meta-analysis of nine randomised trials of selective estrogen receptor modulators (SERMs) indicated women at increased risk of breast cancer had at least a 30% lower risk of the disease if they were allocated to the treatment arm compared with placebo [3]. These trials informed the UK National Institute for Health and Care Excellence (NICE) clinical guideline for familial breast cancer (CG164) which recommended offering preventive therapy (tamoxifen or raloxifene) to women at increased risk of breast cancer [4, 5]. Optimal therapeutic benefit depends on adequate uptake and adherence to the recommended course of therapy. However, uptake can be low and long-term persistence problematic [6–8].

Patient’s decision-making about preventive therapy is complex and emotionally charged. Women may be reluctant to initiate tamoxifen if they have concerns about side effects and perceive uncertain treatment efficacy and low personal risk [9–12]. Family and friends’ experiences of cancer and side effects of medicines may shape women’s beliefs and perceptions about their risk and preventive therapy [13]. There is research to suggest that the decision to undergo prophylactic surgery is influenced by the presence and number of children within the family [14–16] and marital status [17]. Familial networks may influence women’s decision-making regarding preventive therapy use.

Women in the least deprived socioeconomic groups have a higher breast cancer incidence, but lower mortality [1]. Differences in incidence may partly be explained by higher screening uptake among more affluent groups [18, 19]. However, incidence appears to be increasing among ethnic minorities [20]. If preventive therapy is more readily accepted by particular population groups, its introduction could inadvertently create or exacerbate inequalities in breast cancer incidence. Uptake of preventive therapy across different socio-demographic groups should be monitored closely.

The objectives of this mixed-methods study were to (1) assess the uptake of tamoxifen following the introduction of clinical guidelines [4]; (2) evaluate socio-demographic and clinical factors associated with tamoxifen initiation; and (3) undertake an in-depth exploration of women’s experiences of treatment decision-making using qualitative interview data.

## Methods

### Recruitment

In the UK, women present to their primary care physician (general practitioner) to discuss their personal risk of breast cancer, and are referred to secondary care if they are likely to meet breast cancer risk criteria [4, 5]. The type of clinic they are referred to depends on local protocol and strength of their family history. While local variation exists, clinical genetics centres see women at high risk of breast cancer (≥ 30% lifetime risk) where they are counselled about their risk and their eligibility for genetic screening for the breast cancer genes BRCA1 and BRCA2. Family history clinics provide breast screening and risk assessment for women who are at increased risk but not BRCA carriers. Breast clinics who routinely investigate women with suspicious breast lesions may also manage women with a family history. Women with family histories that indicate a moderately high risk (17–30% lifetime risk) will be managed within family history clinics and breast clinics.

In the survey study, women were approached following their appointment at one of the following clinic types; family history clinics (*n* = 12), breast clinics (*n* = 4), clinical genetic centres (*n* = 3) and a family history clinic with genetics support (*n* = 1). Research nurses or clinicians at twenty sites across England recruited participants between September, 2015 and December, 2016. Following verbal consent, participant data were made available to the research team via a secure online portal. Data included: patient age; address and postcode; email address; and risk classification [moderately high risk or high risk]. Participants were assigned a unique study identification code, which was linked to the baseline survey. Participants were invited to complete a baseline survey and return it to the research team using a freepost envelope. Women who did not respond were sent a reminder postcard after 2 weeks, and a full study pack at 4 weeks. Respondents to the baseline questionnaire were sent a follow-up survey at 3 months, and the same approach to reminders was used. In the interview study, women were approached following an appointment at one of two family history clinics.

### Eligibility criteria

Women were eligible to participate in the survey and/or qualitative interviews if they were aged ≥ 18 years, spoke English, had a discussion with a healthcare professional within their appointment about preventive therapy, were classified as having a moderately high or high risk of breast cancer by NICE guidelines, and had no known contraindications for tamoxifen use. Women were excluded if they were unable to consent, read English or had a previous diagnosis of breast cancer.

### Measures

The baseline survey contained the following measures, some of which were dichotomised for analysis: marital status (married/cohabiting vs. single/divorced/separated/widowed); ethnicity (White groups vs. others); education (≥ degree level vs. < degree level); employment (full/part-time vs. all other employment types); and self-reported health (poor, fair, good, excellent). Nulliparity was assessed by the following item: ‘Do you have any children? If so, how many?’ Women were classified as having children or not.

Age was calculated from date of birth provided from NHS records entered onto the secure portal. Women were coded as ≤ 35 years; 36–49 years; and ≥ 50 years. Index of Multiple Deprivation (IMD) scores were calculated from the participant’s postcode, and women were classified into tertiles [21].

Breast cancer risk category (moderately high or high), as outlined by NICE guidelines, was provided by clinic staff [4].

Uptake of tamoxifen was assessed in the 3-month follow-up questionnaire. Women were asked to indicate which of the following 7 statements applied to them: (1) ‘I decided immediately that I did not want to take tamoxifen’; (2) ‘After some thought, I decided that I did not want to take tamoxifen’; (3) ‘I am still deciding if I want to take tamoxifen’; (4) ‘I met with my GP to talk about tamoxifen, and I decided against taking it’; (5) ‘I met with my GP to talk about tamoxifen, but they would not prescribe it’; (6) ‘I have a prescription for tamoxifen from my GP’; and (7) ‘I am currently taking tamoxifen’. Women were classified as initiating tamoxifen if they reported either of the latter two responses.

### Data analysis

The quantitative analyses were pre-registered [22]. Differences in socio-demographic and clinical factors between responders and non-responders to the baseline questionnaire, and between those who completed the baseline survey and women who returned both a baseline and follow-up survey were analysed using Pearson’s Chi-square (*Χ*^2^) tests.

Among women providing outcome data on tamoxifen initiation, Pearson’s *x*^2^ tests were used to compare uptake by participant characteristics. A multivariable logistic regression compared the role of nulliparity on uptake, adjusting for participant characteristics. Clinic type was not included in our analyses due to multicollinearity with breast cancer risk category; those attending clinical genetics centres are likely to have a higher risk of breast cancer. Analysis was undertaken in SPSS v24.0. Statistical significance was set at a 2-sided *p* < 0.05.

During January 2014 to November 2016, face-to-face interviews were conducted to explore women’s breast cancer prevention decision-making. Interviews were digitally recorded, transcribed verbatim, and anonymised. Interview transcripts were coded using the method of constant comparison, whereby data are compared systematically for similarities and differences and coded accordingly [23]. The analysis allowed patterns and relationships between themes to emerge. Overarching themes were developed, which were iteratively refined over the course of analysis. NVivo10 supported the analysis.

## Results

### Survey data

In total, 732 women were invited to complete a survey. Baseline surveys were returned by 408 women (55.7%). Among the 408 respondents, 275 surveys (67.4%) were returned by women attending family history clinics, 59 surveys (14.5%) were from a combined family history and genetic centre; 49 surveys (12%) were from breast clinics and 25 (6.1%) from clinical genetic centres. There were no differences between responders and non-responders with regard to clinical risk (*p* = 0.62), socioeconomic status (*p* = 0.054) or age group (*p* = 0.086) (Table S1). Data on the decision to initiate chemoprevention were provided by 258 (63.2%) of previous respondents at least 3 months after the initial appointment. Women were more likely to provide follow-up data if they were from a higher socioeconomic status group (*p* < 0.001). There were no other socio-demographic differences between women who provided baseline data only and those who returned a 3-month survey (Table S2).

Women providing data at both time points (*n* = 258) are described in Table 1. The mean age was 45.4 years (SD = 7.89); 10.1% were aged ≤ 35 years old; 65.1% were aged 36–49 years; and 24.8% were ≥ 50 years old. The majority of women had children (79.5%), were white (96.5%), educated below degree level (54.7%), married or cohabiting (77.0%) and were in full-time employment (85.7%). Most women reported having either ‘good’ (59.0%) or ‘excellent’ (18.4%) health. There were more women at moderately high risk of breast cancer (61.6%) than high risk (37.6%).

**Table 1:** Uptake of breast cancer chemoprevention by participant characteristics and multivariable logistic regression model (*n* = 258)

	N (%)	Uptake (%)	OR (95% CI)	*p* value
Children				
Yes	205 (79.5)	17.6	5.26 (1.13, 24.49)	0.035
No	53 (20.5)	3.8	Ref	Ref
Ethnic group				
White	247 (96.5)	15.0	1.24 (0.13, 12.32)	0.853
Other	9 (3.5)	11.1	Ref	Ref
Education level				
Degree or above	116 (45.3)	17.2	1.81 (0.85, 3.86)	0.124
Below degree level	140 (54.7)	12.9	Ref	Ref
Health status				
Poor^†^	11 (4.3)	0	–	–
Fair	47 (18.4)	10.6	0.83 (0.23, 2.99)	0.774
Good	151 (59.0)	16.6	1.28 (0.49, 3.37)	0.611
Excellent	47 (18.4)	14.9	Ref	Ref
Risk level				
Moderate	159 (61.6)	15.1	0.93 (0.44, 1.98)	0.856
High	97 (37.6)	14.4	Ref	Ref
Unclear^†^	2 (0.8)	0	–	–
SES				
Low (most deprived)	59 (23.2)	11.9	1.13 (0.41, 3.09)	0.815
Middle	86 (33.9)	16.3	1.58 (0.67, 3.71)	0.299
High (least deprived)	109 (42.9)	14.7	Ref	Ref
Employment				
Full-time	221 (85.7)	14.5	Ref	Ref
All other employments	37 (14.3)	16.2	1.46 (0.53, 4.01)	0.462
Marital status				
Married or cohabiting	198 (77.0)	16.7	1.71 (0.53, 5.46)	0.366
Unmarried	59 (23.0)	8.5	Ref	Ref

^†^category not included in multivariable analyses due to insufficient cases. Numbers may not round to 258 due to missing data. The multivariable model included 235 respondents

Uptake of chemoprevention was 14.7%. Women who had children were more likely to initiate chemoprevention than those without children (17.6% vs. 3.8%, respectively). Differences in uptake did not vary between other socio-demographic groups. In a multivariable model adjusting for participant characteristics, women who had children were significantly more likely to have initiated chemoprevention than those without children, although confidence intervals were wide (OR = 5.26, 95% CI = 1.13, 24.49, *p* = 0.035) (Table 1). No other factors affected uptake of chemoprevention in the multivariable model. Uptake of chemoprevention by age group was 3.8% for women aged ≤ 35 years; 17.3% for women aged 36–49 years; and 12.5% for women aged ≥ 50 years. Age was not entered into the regression model, due to the small number of women aged ≤ 35 years initiating chemoprevention. Quantitative observations were explored in the qualitative analysis.

### Interview data

In total, 18 women returned consent forms and 16 completed a semi-structured interview (Table 2). Average interview length was 35 min (range 20–53 min).

**Table 2:** Interview participant characteristics

Pseudonym	Age (years)	Marital status	Ethnicity	Self-reported risk	Number of children
SD	44	Single	White British	Moderate	0
MC	60	Married	White British	Moderate	2
TT	49	Single	White British	High	0
PO	52	Single	White British	Moderate	0
LM	53	Cohabiting	White British	Moderate	0
AS	53	Married	White British	High	2
CD	57	Cohabiting	Greek Cypriot	Unsure	0
JP	35	Married	Asian	Moderate	2
PL	46	Married	White British	Unsure	3
LL	47	Married	White British	Moderate	0
RF	26	Single	Asian British	Moderate	0
VI	40	Cohabiting	White British	Moderate	0
EK	48	Single	White British	High	0
YN	59	Cohabiting	White British	High	2
JB	38	Married	White British	Unsure	2
KU	45	Divorced/separated	White British	High	2

The interviews confirmed that the decision to initiate preventive therapy is affected by a multitude of psychological and social factors. Decision-making was heavily influenced by family and close friends. The following themes were important to the decision-making process.

## Theme 1: Considering children in making decisions

For some women, having children had a major influence on their consideration of the advantages and disadvantages of taking tamoxifen. The women described how they thought of their children, and not just themselves, when considering cancer prevention and risk.

…If it reduces my chances of developing breast cancer, I am healthier for [my children], I am around for them… So I feel that it just makes me more secure as a parent…All of a sudden, my health is more important because it affects them as well as me. (JP, 35 years, married, 2 children).

These women were aware of the possible side effects of tamoxifen, but consideration of their children weighed stronger. Consequently they were more willing to consider taking tamoxifen.

I’m not necessarily going to get breast cancer, but if it can prevent it, I would be willing to take it, definitely. Obviously I’ve got young children to think about now. I would be happy to start taking it, but yes, I do look at the side effects. (JB, 38 years, married, 2 children).

However, one woman felt the immediate impact on her quality of life would have consequences for her family and children. YN was concerned about how side effects would impinge on her ability to care for her parents and children, and therefore did not feel like she could cope with any additional burden in the short-term.

I have huge responsibilities to the point where I’ve had to stop working, and so I need to try and be as healthy as I can and function as best I can. If I’m going to go on a medication… which okay maybe in the long term might be beneficial, possibly…but in the short term I just can’t cope…I’ve weighed up the pros and cons and…don’t want to go down that route. (YN, 59 years, cohabiting, 2 children).

## Theme 2: Impact of others’ on beliefs about medication

Familial networks influenced women’s attitudes and beliefs towards taking medication in general. A culture of stoicism and distrust of medications within the family resulted in more negative attitudes and beliefs towards tamoxifen uptake.

I come from a family of people who don’t take tablets, and who definitely don’t take tablets on a regular basis. “They’re bad”, that’s the general feeling about tablets. There’s something about willpower in it as well, “you don’t need to take a tablet. You just need to grit your teeth and get through it”. (LL, 47 years, married, 0 children).

Women who reported negative attitudes towards medication within their social networks felt their family would hold strong views about tamoxifen use. This impacted their likelihood of communicating with family members around taking tamoxifen.

I don’t know exactly the amount of cancer within the family because in those days as well what you’ve got to remember is it was the c word…I haven’t really discussed [tamoxifen] with anybody to be honest. (LM, 53 years, cohabiting, 0 children).

These women were also more likely to maintain a stoical attitude, discussing their reluctance to take medications in general on a regular basis.

I don’t like taking medication. I will avoid taking medications because I think that’s part of my philosophy. I’m not an interventionist, I think I have a fear that taking medications may impact on your body in negative ways…we don’t always understand all of the impact that chemicals may have on our body. (MC, 60 years, married, 2 children).

Women who witnessed significant others adhering to daily medication regimens for other conditions had more positive attitudes and beliefs towards tamoxifen than those without these social models.

My dad is on quite a lot of medication for high blood pressure, and diabetes. I’ve grown up with him always being on it, so it doesn’t really feel any different…to have someone who’s on medication…It wouldn’t impact my life, but it would be a different routine to have to get used to. (RF, 26 years, single, 0 children).

Many women did not associate tamoxifen with cancer prevention. Instead, they considered it as a cancer treatment, which evoked fear and painful memories about significant others.

She used the word “tamoxifen” and I think that frightened me. I knew what that word meant. That word meant cancer, not chemoprevention. (LL, 47 years, married, 0 children).

All women sought the opinion of significant others, particularly female family members, who had experience of taking tamoxifen as an adjuvant treatment. Male partners were rarely consulted. These discussions often negatively influenced women’s attitudes towards initiating preventive therapy, particularly with regard to side effects.

I spoke to my best friend about it actually and she said, “No way because maybe it’d give you the menopause”. She said she wouldn’t take it herself but she said, “No don’t take it either because what would you want to give yourself that for? (TT, 49 years, single, 0 children).

Women questioned the value of taking preventive therapy if it had not been effective for a close family member taking tamoxifen as part of their treatment for breast cancer.

Another thing that has swung my decision around not taking tamoxifen is that when my mother had breast cancer she was prescribed tamoxifen at one point…and it didn’t have any effect for her at all…If your mother’s cancer didn’t respond to it, then I would expect that I wouldn’t respond either and therefore it may not give me any additional protection. (MC, 60 years, married, 2 children).

## Theme 3: Emotional response to risk

Following their consultation, some women were confronted with feelings of fear, anxiety and denial. They reported a lack of control over their cancer risk, and the options available to them. For many, their upbringing and significant others were the driver of these emotions.

Cancer, the actual word cancer wasn’t even used, my mum came from a very Irish farm background where things like that were not discussed. (LM, 53 years, cohabiting, 0 children).

Some women felt they were predestined to develop cancer, due to multiple family members developing and dying from cancer. They expressed a diminished sense of responsibility, where they had little control over their breast cancer risk. This negatively influenced decisions about tamoxifen uptake.

I always felt…I would develop breast cancer. I was eight when [my mother] died…It’s very magical thinking about, well, if I’m going to get it, I’m going to get it, and it doesn’t matter what I do. That it’s kind of written in the stars…that it’s my fate. So, what then would have been the point of any breast cancer prevention…? (LL, 47 years, married, 0 children).

## Discussion

In this multicentre study investigating uptake of tamoxifen following the introduction of UK clinical guidelines [4], only one in seven women at increased risk of breast cancer initiated preventive therapy. There were no socio-demographic differences in uptake, indicating inequalities are unlikely to be created or exacerbated by the introduction of preventive therapy into routine clinical practice. However, this observation does not preclude the potential for inequalities to be occurring at a different point in the clinical pathway, such as presentation to primary care for risk assessment referral. There is evidence that people from an ethnic minority background are less likely to access cancer genetic services [24]. The low level of uptake reported here is comparable with previous meta-analyses reporting 9% [7] and 15% [25] of women accepting preventive therapies in non-trial settings. Similar data were reported in a UK single centre study undertaken in the months preceding the release of NICE guideline CG164 [13]. The factors influencing uptake of preventive therapy are poorly understood, which hampers our ability to support informed patient decision-making and optimise therapeutic benefit [7]. We demonstrated that the complex decision to initiate tamoxifen does not occur in isolation; choosing to initiate a medication is considered within the context of social, work and family commitments [26, 27].

Perceptions of personal need for medication and concerns about its negative effects are key to decision-making [28, 29]. Having children increased the perceived need for preventive therapy; women were more likely to initiate tamoxifen if they had children because a reduction in risk meant they would be healthier for longer and thereby more able to care for their families. As a result, having children weighed stronger than experiencing side effects of medications. However, the decision to initiate tamoxifen remained unresolved if women expressed strong concerns about preventive therapies. Some women decided not to initiate tamoxifen because of concern about the impact possible side effects would have on their caregiving roles. Preventive therapy decision-making should be considered within the context of women’s short- and long-term goals and priorities, such as family, social or work commitments [30].

Distrust of medicines and a culture of stoicism within the family influenced women’s beliefs towards taking regular medication and reduced the likelihood of discussing tamoxifen with family members. This demonstrates how family and friend’s experiences contribute to women’s beliefs towards their breast cancer risk and the use of preventive therapies. Although individuals’ beliefs about disease and treatment are amenable to change within a healthcare setting [31], it is important to recognise that beliefs and feelings are constructed and embedded within the social context.

This study had limitations. As with most observational studies there is a risk of selection bias in response and retention rates. Confidence intervals were wide for the association between having children and tamoxifen uptake. Although tamoxifen was the most commonly offered preventive therapy, raloxifene may have been discussed in some centres. This did not appear to influence uptake in those centres. All women were given three months to decide whether they would like to initiate tamoxifen, however, some women may not have made a decision at the time of returning the survey. Higher uptake may be seen with longer follow-up. These data were from surveys which are reliant on accurate self-reporting.

In conclusion, uptake of tamoxifen in women at increased risk of breast cancer is low in routine clinical practice. There were no socio-demographic differences in uptake, which is encouraging for the future adoption of other preventive therapy strategies. However, this observation does not preclude other potential sources of inequality, such as primary care presentation. Women weigh up the risks and benefits of preventive therapies within the context of other commitments and priorities, including their family and children. Encouraging women to discuss their beliefs and perceptions about preventive therapies within the consultation may help support informed decision-making.

## Data availability

Researchers can apply for access to these data by contacting the corresponding author.

## Electronic supplementary material

Below is the link to the electronic supplementary material.

Supplementary material 1 (DOC 62 kb)

## References

[1] Cancer Research UK (2015) Breast cancer statistics. http://www.cancerresearchuk.org/health-professional/cancer-statistics/statistics-by-cancer-type/breast-cancer. Accessed Feb 2018

[2] Smittenaar CR, Petersen KA, Stewart K, Moitt N (2016). Cancer incidence and mortality projections in the UK until 2035. Br J Cancer.

[3] Cuzick J, Sestak I, Bonanni B, Costantino JP, Cummings S, DeCensi A, Dowsett M, Forbes JF, Ford L, LaCroix AZ, Mershon J, Mitlak BH, Powles T, Veronesi U, Vogel V, Wickerham DL (2013). Selective oestrogen receptor modulators in prevention of breast cancer: an updated meta-analysis of individual participant data. Lancet.

[4] National Institute for Health and Care Excellence (NICE) (2013) Familial breast cancer (CG164): Classification and care of people at risk of familial breast cancer and management of breast cancer and related risks in people with a family history of breast cancer. Issued: June. https://www.nice.org.uk/guidance/cg164. Accessed Feb 2018 31909932

[5] National Institute for Health and Care Excellence (NICE) (2017) Familial breast cancer (CG164): Classification, care and managing breast cancer and related risks in people with a family history of breast cancer. Issued: June. https://www.nice.org.uk/guidance/cg164. Accessed Feb 2018

[6] Sestak I, Smith SG, Howell A, Forbes JF, Cuzick J (2018). Early participant-reported symptoms as predictors of adherence to anastrozole in the International Breast Cancer Intervention Studies II. Ann Oncol.

[7] Smith SG, Sestak I, Forster A, Partridge A, Side L, Wolf MS, Horne R, Wardle J, Cuzick J (2016). Factors affecting uptake and adherence to breast cancer chemoprevention: a systematic review and meta-analysis. Ann Oncol.

[8] Smith SG, Sestak I, Howell A, Forbes J, Cuzick J (2017). Participant-reported symptoms and their effect on long-term adherence in the International Breast Cancer Intervention Study I (IBIS I). J Clin Oncol.

[9] Altschuler A, Somkin CP (2005). Women’s decision making about whether or not to use breast cancer chemoprevention. Women Health.

[10] Bober SL, Hoke LA, Duda RB, Regan MM, Tung NM (2004). Decision-making about tamoxifen in women at high risk for breast cancer: clinical and psychological factors. J Clin Oncol.

[11] Heisey R, Pimlott N, Clemons M, Cummings S, Drummond N (2006). Women’s views on chemoprevention of breast cancer: qualitative study. Can Fam Physician.

[12] Salant T, Ganschow PS, Olopade OI, Lauderdale DS (2006). Why take it if you don’t have anything? breast cancer risk perceptions and prevention choices at a public hospital. J Gen Intern Med.

[13] Donnelly LS, Evans DG, Wiseman J, Fox J, Greenhalgh R, Affen J, Juraskova I, Stavrinos P, Dawe S, Cuzick J, Howell A (2014). Uptake of tamoxifen in consecutive premenopausal women under surveillance in a high-risk breast cancer clinic. Br J Cancer.

[14] Lostumbo L, Carbine NE, Wallace J (2010). Prophylactic mastectomy for the prevention of breast cancer. Cochrane Database Syst Rev.

[15] Skytte AB, Gerdes AM, Andersen MK, Sunde L, Brøndum-Nielsen K, Waldstrøm M, Kølvraa S, Crüger D (2010). Risk-reducing mastectomy and salpingo-oophorectomy in unaffected BRCA mutation carriers: uptake and timing. Clin Genet.

[16] Stuckey A, Dizon D, Wilbur JS, Kent J, Tejada-Berges T, Gass J, Legare R (2010). Clinical characteristics and choices regarding risk-reducing surgery in BRCA mutation carriers. Gynecol Obstet Invest.

[17] Howard-McNatt M, Schroll RW, Hurt GJ, Levine EA (2011). Contralateral prophylactic mastectomy in breast cancer patients who test negative for BRCA mutations. Am J Surg.

[18] Maheswaran R, Pearson T, Jordan H, Black D (2006). Socioeconomic deprivation, travel distance, location of service, and uptake of breast cancer screening in North Derbyshire, UK. J Epidemiol Community Health.

[19] Renshaw C, Jack RH, Dixon S, Møller H, Davies EA (2010). Estimating attendance for breast cancer screening in ethnic groups in London. BMC Public Health.

[20] Stotter A, Jenkins J, Edmondson-Jones M, Blackledge H, Kearins O (2014). Temporal changes in breast cancer incidence in South Asian women. Cancer Epidemiol.

[21] McLennan D, Barnes H, Noble M, Davies J, Garratt E, Dibben C (2011) The English indices of deprivation 2010. Department for Communities and Local Government, March 2011. https://www.gov.uk/government/uploads/system/uploads/attachment_data/file/6320/1870718.pdf. Accessed Feb 2018

[22] Smith S (2017) ENGAGE study. https://osf.io/ud67j

[23] Glaser BG, Strauss AL (1967). The discovery of grounded theory.

[24] Jacobs C, Rawson R, Campion C, Caulfield C, Heath J, Burton C, Kavalier F (2007). Providing a community-based cancer risk assessment service for a socially and ethnically diverse population. Fam Cancer.

[25] Ropka ME, Keim J, Philbrick JT (2010). Patient decisions about breast cancer chemoprevention: a systematic review and meta-analysis. J Clin Oncol.

[26] Hallowell N, Jacobs I, Richards M, Mackay J, Gore M (2001). Surveillance or surgery? a description of the factors that influence high risk premenopausal women’s decisions about prophylactic oophorectomy. J Med Genet.

[27] Pound P, Britten N, Morgan M, Yardley L, Pope C, Daker-White G, Campbell R (2005). Resisting medicines: a synthesis of qualitative studies of medicine taking. Soc Sci Med.

[28] Horne R, Chapman SCE, Parham R, Freemantle N, Forbes A, Cooper V (2013). Understanding patients’ adherence-related beliefs about medicines prescribed for long-term conditions: a meta-analytic review of the necessity-concerns framework. PLoS ONE.

[29] Horne R, Weinman J (1999). Patients’ beliefs about prescribed medicines and their role in adherence to treatment in chronic physical illness. J Psychosom Res.

[30] Thorneloe RJ, Bundy C, Griffiths CEM, Ashcroft DM, Cordingley L (2017). Nonadherence to psoriasis medication as an outcome of limited coping resources and conflicting goals: findings from a qualitative interview study with people with psoriasis. Br J Dermatol.

[31] Phillips LA, Leventhal H, Leventhal EA (2012). Physicians’ communication of the common-sense self-regulation model results in greater reported adherence than physicians’ use of interpersonal skills. Br J Health Psychol.

