# Supplementary material for: Uptake of breast cancer preventive therapy in the UK: results from a multicentre prospective survey and qualitative interviews
Source: Breast Cancer Res Treat. 2018 Apr 24;170(3):633–40. doi: 10.1007/s10549-018-4775-1 (PMC6022517; doi:10.1007/s10549-018-4775-1)
Supplement: Supplementary file 1 — Supplementary material 1 (DOC 62 kb) [file 10549_2018_4775_MOESM1_ESM.doc]

| **Table S1. Univariable comparison of response by participant characteristics (n=732)** | | | |
| --- | --- | --- | --- |
|  | **Non-responders** | **Responders** | **p-value** |
|  | **N (%)** | **N (%)** |  |
| **Risk** |  |  |  |
| Moderate | 202 (45.4) | 243 (54.6) | 0.62 |
| High | 118 (42.6) | 159 (57.4) |  |
| Unclear | 4 (40) | 6 (60) |  |
| **SES** |  |  |  |
| Low (most deprived) | 115 (48.9) | 120 (51.1) | 0.054 |
| Middle | 105 (44.5) | 131 (55.5) |  |
| High (least deprived) | 92 (38.0) | 150 (62.0) |  |
| **Age** |  |  |  |
| ≤ 35 years | 43 (51.2) | 41 (48.8) | 0.086 |
| 36-49 years | 213 (45.1) | 259 (54.9) |  |
| ≥ 50 years | 65 (37.6) | 108 (62.4) |  |

**SUPPLEMENTARY FILES**

Numbers may not round to 732 due to missing data.

| **Table S2. Univariable comparison of retention by participant characteristics (n=408)** | | | |
| --- | --- | --- | --- |
|  | **Baseline only** | **Baseline + 3 months** | **p-value** |
|  | **N (%)** | **N (%)** |  |
| **Risk** |  |  |  |
| Moderate | 84 (34.6) | 159 (65.4) | 0.21 |
| High | 62 (39.0) | 97 (61.0) |  |
| Unclear | 4 (66.7) | 2 (33.3) |  |
| **SES** |  |  |  |
| Low (most deprived) | 61 (50.8) | 59 (49.2) | <0.001 |
| Middle | 45 (34.4) | 86 (65.6) |  |
| High (least deprived) | 41 (27.3) | 109 (72.7) |  |
| **Marital status** |  |  |  |
| Married or cohabiting | 100 (33.6) | 198 (66.4) | 0.095 |
| Unmarried | 44 (42.7) | 59 (57.3) |  |
| **Ethnic group** |  |  |  |
| White | 137 (35.7) | 247 (64.3) | 0.217 |
| Other | 9 (50.0) | 9 (50.0) |  |
| **Education level** |  |  |  |
| Degree or above | 60 (34.1) | 116 (65.9) | 0.556 |
| Below degree level | 82 (36.9) | 140 (63.1) |  |
| **Employment** |  |  |  |
| Employed | 127 (36.5) | 221 (63.5) | 0.785 |
| Unemployed | 23 (38.3) | 37 (61.7) |  |
| **Health status** |  |  |  |
| Poor | 5 (31.3) | 11 (68.8) | 0.523 |
| Fair | 31 (39.7) | 47 (60.3) |  |
| Good | 89 (37.1) | 151 (62.9) |  |
| Excellent | 19 (28.8) | 47 (71.2) |  |
| **Age** |  |  |  |
| ≤ 35 years | 15 (36.6) | 26 (63.4) | 0.597 |
| 36-49 years | 91 (35.1) | 168 (64.9) |  |
| ≥ 50 years | 44 (40.7) | 64 (59.3) |  |

Numbers may not round to 408 due to missing data.
